# Cellular hormetic response to 27-hydroxycholesterol promotes neuroprotection through AICD induction of MAST4 abundance and kinase activity

**DOI:** 10.1038/s41598-017-13933-9

**Published:** 2017-10-24

**Authors:** Brendan Gongol, Traci L. Marin, John D. Jeppson, Karina Mayagoitia, Samuel Shin, Nicholas Sanchez, Wolff M. Kirsch, Harry V. Vinters, Christopher G. Wilson, Othman Ghribi, Salvador Soriano

**Affiliations:** 1 Department of Pathology and Human Anatomy, Division of Anatomy, Loma Linda University School of Medicine, Loma Linda, CA 92350 USA; 2 Cardiopulmonary Sciences, Schools of Allied Health Professions and Medicine, Loma Linda University, Loma Linda, CA 92350 USA; 3 Department of Basic Sciences, Division of Biochemistry, School of Medicine, Loma Linda University, Loma Linda, CA 92350 USA; 4 Section of Neuropathology, Ronald Reagan UCLA Medical Center, David Geffen School of Medicine at UCLA, Los Angeles, 90095 USA; 5 Department of Basic Sciences, Division of Physiology, School of Medicine, Loma Linda University, Loma Linda, CA 92350 USA; 6 Department of Basic Sciences, School of Medicine and Health Sciences, University of North Dakota, Grand Forks, ND 58202 USA

## Abstract

The function of the amyloid precursor protein (APP) in brain health remains unclear. This study elucidated a novel cytoprotective signaling pathway initiated by the APP transcriptionally active intracellular domain (AICD) in response to 27-hydroxycholesterol (27OHC), an oxidized cholesterol metabolite associated with neurodegeneration. The cellular response to 27OHC was hormetic, such that low, but not high, doses promoted AICD transactivation of microtubule associated serine/threonine kinase family member 4 (MAST4). MAST4 in turn phosphorylated and inhibited FOXO1-dependent transcriptional repression of rhotekin 2 (RTKN2), an oxysterol stress responder, to optimize cell survival. A palmitate-rich diet, which increases serum 27OHC, or APP ablation, abrogated this response *in vivo*. Further, this pathway was downregulated in human Alzheimer’s Disease (AD) brains but not in frontotemporal dementia brains. These results unveil MAST4 as functional kinase of FOXO1 in a 27OHC AICD-driven, hormetic pathway providing insight for therapeutic approaches against cholesterol associated neuronal disorders.

## Introduction

The biological function of the amyloid precursor protein (APP) as a signaling molecule in the brain remains unresolved, presenting a significant hurdle to understand its role in late-onset Alzheimer’s disease (AD) etiology^1–3^. APP counters AD pathogenic triggers, such as oxidative stress, inflammation, and dyslipidemia, by transducing signals initiated by extracellular cholesterol stimuli to establish and maintain cellular cholesterol homeostasis^3–6^. This downstream signaling is dependent upon the function of APP cleavage products, including the transcriptionally active amyloid intracellular domain (AICD)^7,8^. The importance of APP signaling in cholesterol homeostasis and survival is demonstrated by expedited mortality in APP and Niemann-Pick disease type C (NPC) double knock out mice (*APP* ^*−/−*^ */NPC* ^*−/−*^) compared to *NPC* ^*−/−*^ mice^9^, which have a short life span due to aberrant cholesterol storage and transport mechanisms.

The aim of this study was to determine whether APP regulates a physiological response to 27-hydroxycholesterol (27OHC), an oxidized cholesterol metabolite that accumulates in late-onset AD brain^10^, and whether such a response could be mechanistically linked to late-onset AD. This report demonstrates that APP is necessary to facilitate a response to low dose (5 µM) 27OHC. In response to low doses of 27OHC, the APP intracellular domain (AICD) transactivated microtubule associated serine/threonine kinase family member 4 (MAST4), which phosphorylated and inhibited forkhead box protein O1 (FOXO1)-dependent transcriptional repression of rhotekin 2 (RTKN2), an oxysterol stress responder, to optimize cell survival^11^. Aberrant APP signaling epistatically activates FOXO1 inducing cell death in Alzheimer’s disease (AD) mouse models^12,13^. At higher doses of 27OHC (50 µM), this AICD-driven pathway was inhibited, resulting in decreased abundance of RTKN2. A similar inhibitory response was observed in brains of mice fed a palmitate-rich diet. The AICD-MAST4 protective pathway was impaired in the brains of human late-onset AD, but not frontotemporal dementia (FTD) subjects, a neurodegenerative disease associated with genetic risk factors and unknown environmental causes^14^.

## Results

### APP mediates a hormetic response to 27OHC

**Figure 1:**
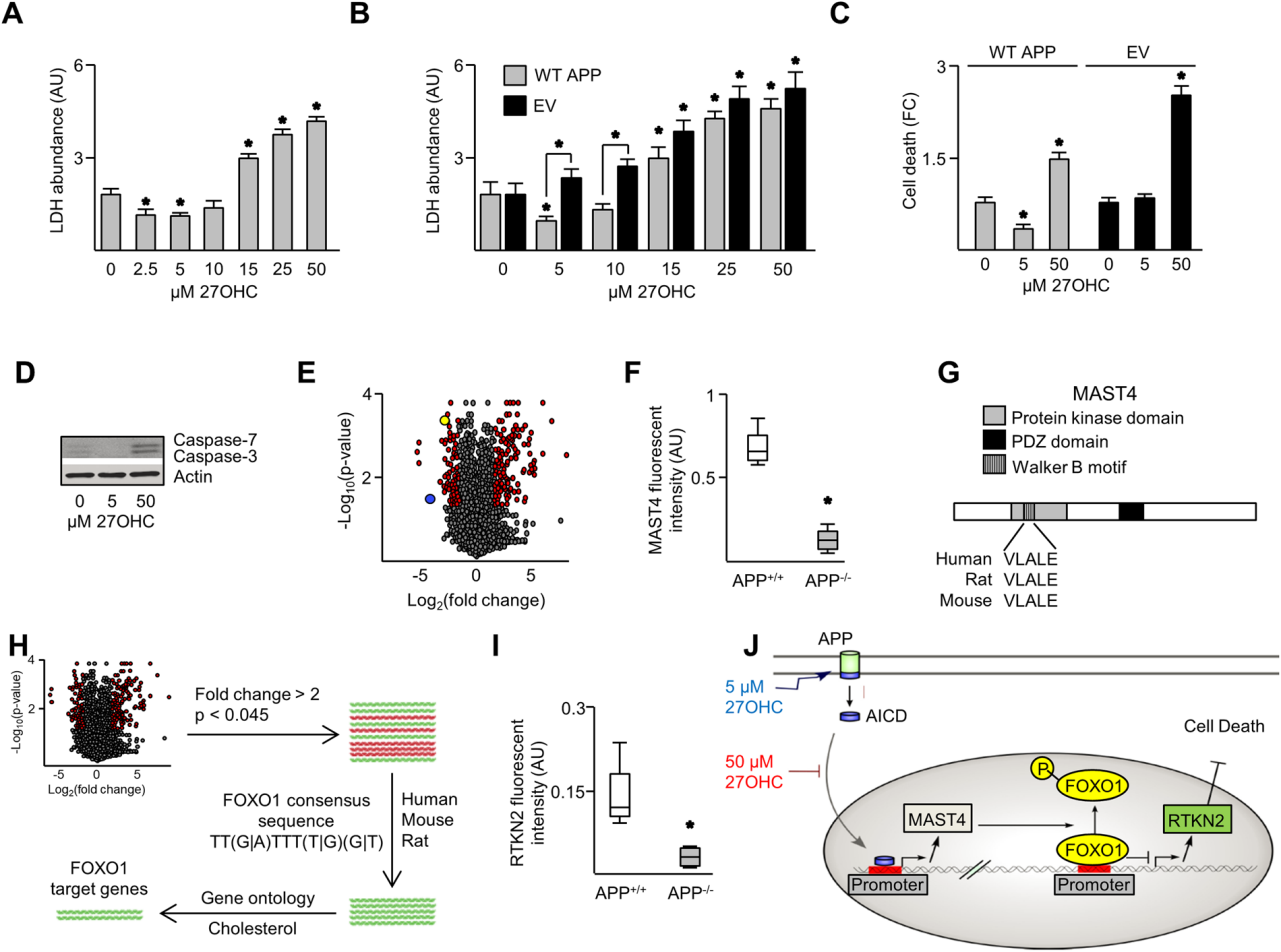
27OHC elicited a hormetic response through APP. Secreted LDH abundance from nd-SH-SY5Y cells (**A**) or B103 cells transfected with WT APP or empty vector (EV) (**B**), treated with 27OHC for 18 hours. (**C**) Live/dead analysis of B103 cells transfected with indicated vectors and treated as indicated. (**D**) Immunoblot of Caspases-7 and −3 in nd-SH-SY5Y cells treated as indicated. (**E**) Volcano plot of microarray data illustrating APP^*−*/*−*^/APP^+/+^ genotype fold changes in mouse brain cortex. Points in red represent genes with a fold change greater than 2 and a p-value less than 0.045. MAST4 is represented in yellow and RTKN2 in blue. All other points are illustrated in black. (**F**) Raw MAST4 mRNA abundance in microarray data. (**G**) Protein domains of MAST4 and Walker B sequence. (**H**) Screening strategy for the identification of FOXO1 regulated promoters. (**I**) Raw RTKN2 mRNA abundance in microarray data. (**J**) Illustrated hypothesis for the effects of 5 or 50 µM 27OHC on APP, MAST4, FOXO1, and RTKN2. (**A–F** & **I**) N = 3 independent experiments. *P < 0.05 significance is in comparison to 0 µM 27OHC treatment (**A–B**), untreated WT APP transfected cells (**C**), or the APP^+/+^ genetic background (**F** & **I**).

Secreted lactate dehydrogenase (LDH) is a determinant of cell viability^15^. In neuron-differentiated SH-SY5Y (nd-SH-SY5Y) cells, 27OHC elicited a dose-dependent, hormetic response^16^. Low doses (2.5 to 5 µM) decreased LDH, but high doses (above 15 µM) increased LDH compared to control (Fig. 1A). 24-hydroxycholesterol (24OHC), an oxysterol control^17^, had no effect on LDH (Fig. S1A). LDH secreted from APP null rat central neurons (B103 cells) transfected with wild-type (WT) APP was lower than in cells transfected with empty vector (EV) treated with 27OHC (Fig. 1B). Consistently, dead/live cell assays demonstrated that 5 µM 27OHC was protective in APP-expressing but not EV transfected B103 cells (Fig. 1C). The cytoprotective response to 5 µM 27OHC was confirmed by the expression of activated caspases 3 and 7 proteins^18^ in nd-SH-SY5Y cells (Fig. 1D).

To elucidate the mechanistic pathways involved in APP regulated 27OHC cell viability, transcriptomes were generated and the expression profiles from cerebral cortices of 3-week old *APP* ^+/+^ and *APP* ^*−/−*^ mice were analyzed (Fig. 1E). MAST4 mRNA was significantly decreased in the *APP* ^*−/−*^ brains (Fig. 1F). Sequence analysis of MAST4 revealed a serine/threonine kinase domain containing a walker-B motif with an adjacent aspartic residue, a chemical signature required for kinase activity (Figs 1G, S1B–S1C)^19,20^. Pathway analysis predicted MAST4 association with FOXO1 (Fig. S2A), a stress responder important for lipid metabolism, which is dysregulated in AD^12,13,21^. FOXO1 consensus sequence mapping of cholesterol responsive gene promoters in the transcriptome dataset identified *RTKN2* promoter as a FOXO1 target with species preservation (Fig. 1H). Supporting the role of FOXO1 as a RTKN2 transcriptional repressor, RTKN2 was decreased in *APP* ^*−/−*^ compared to *APP* ^+/+^ cerebral cortical transcriptomes (Fig. 1I). RTKN2 expression is necessary to elicit a cell stress response to oxysterol cytotoxicity, consistent with a role in oxysterol signaling in the brain^11^. Based on these data, a working model was generated for an APP-driven hormetic response to 27OHC (Fig. 1J), proposing that low doses, but not high doses, of 27OHC elicit an AICD-driven transactivation of MAST4, leading to phosphorylation and inhibition of FOXO1 transcriptional repression of RTKN2 to optimize cell survival.

### AICD regulates MAST4 in response to 27OHC

**Figure 2:**
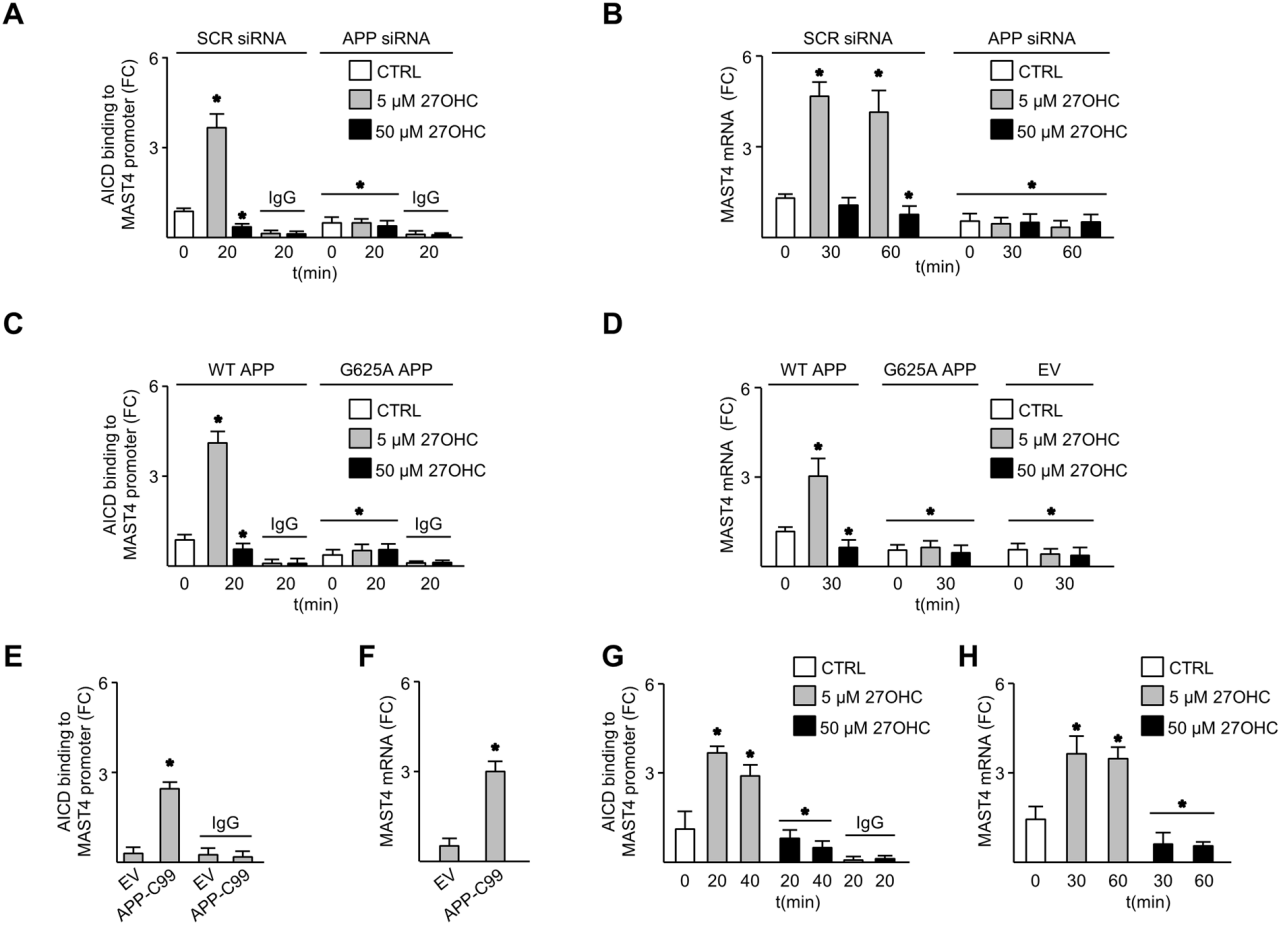
AICD increased expression of MAST4 in response to 27OHC. AICD binding to the *MAST4* promoter (**A**), and MAST4 mRNA abundance (**B**) in nd-SH-SY5Y cells transfected with SCR or APP siRNA. AICD binding to *MAST4* promoter (**C**), and MAST4 mRNA abundance (**D**) in B103 cells transfected with WT, G625A APP, or empty vector (EV) and treated with 27OHC or left untreated. AICD binding (**E**) and MAST4 mRNA (**F**) in B103 cells transfected with APP-C99 or EV. AICD binding to the MAST4 promoter (**G**), and MAST4 mRNA abundance (**H**) in rat cortical (RC) neurons treated with 5 or 50 µM 27OHC. ChIP and mRNA abundance are represented as fold change measurements (FC). (**A–H**) N = 3 independent experiments. *P < 0.05 significance is in comparison to the untreated control group of the SCR siRNA (**A–B**), WT APP (**C–D**), or EV transfected cells (**E**–**F**), or the non-manipulated control group (**G,H**).

Chromatin immunoprecipitation assays demonstrated enhanced AICD association with the *MAST4* promoter to increase MAST4 mRNA and protein abundance in nd-SH-SY5Y cells treated with 5, but not 50 µM 27OHC. Loss of the 27OHC response was observed upon APP siRNA transfection (Figs 2A,B, S3A–C). This was not observed in B103 cells expressing a G625A mutation in the APP cholesterol-sensing domain (Fig. 2C,D). The G625A mutation did not change APP protein abundance but abrogated the ability of APP to bind cholesterol and mobilize to lipid rafts (S3D-E)^22^. Additionally, AICD binding to and transcriptionally activating MAST4 was demonstrated in B103 cells overexpressing the C-terminal fragment of APP that increases AICD abundance (APP-C99) (Fig. 2E,F)^23^. Further, AICD interacted with the *MAST4* promoter in rat cortical (RC) neurons to increase MAST4 mRNA in response to 5 but not 50 µM 27OHC (Fig. 2G,H). These data demonstrate that AICD binding to the *MAST4* promoter to increase MAST4 mRNA and protein abundance is enhanced at 5 while inhibited at 50 µM 27OHC. This signaling event is absent upon mutagenesis of the APP cholesterol binding domain.

### MAST4 phosphorylation of FOXO1 regulates RTKN2 expression

**Figure 3:**
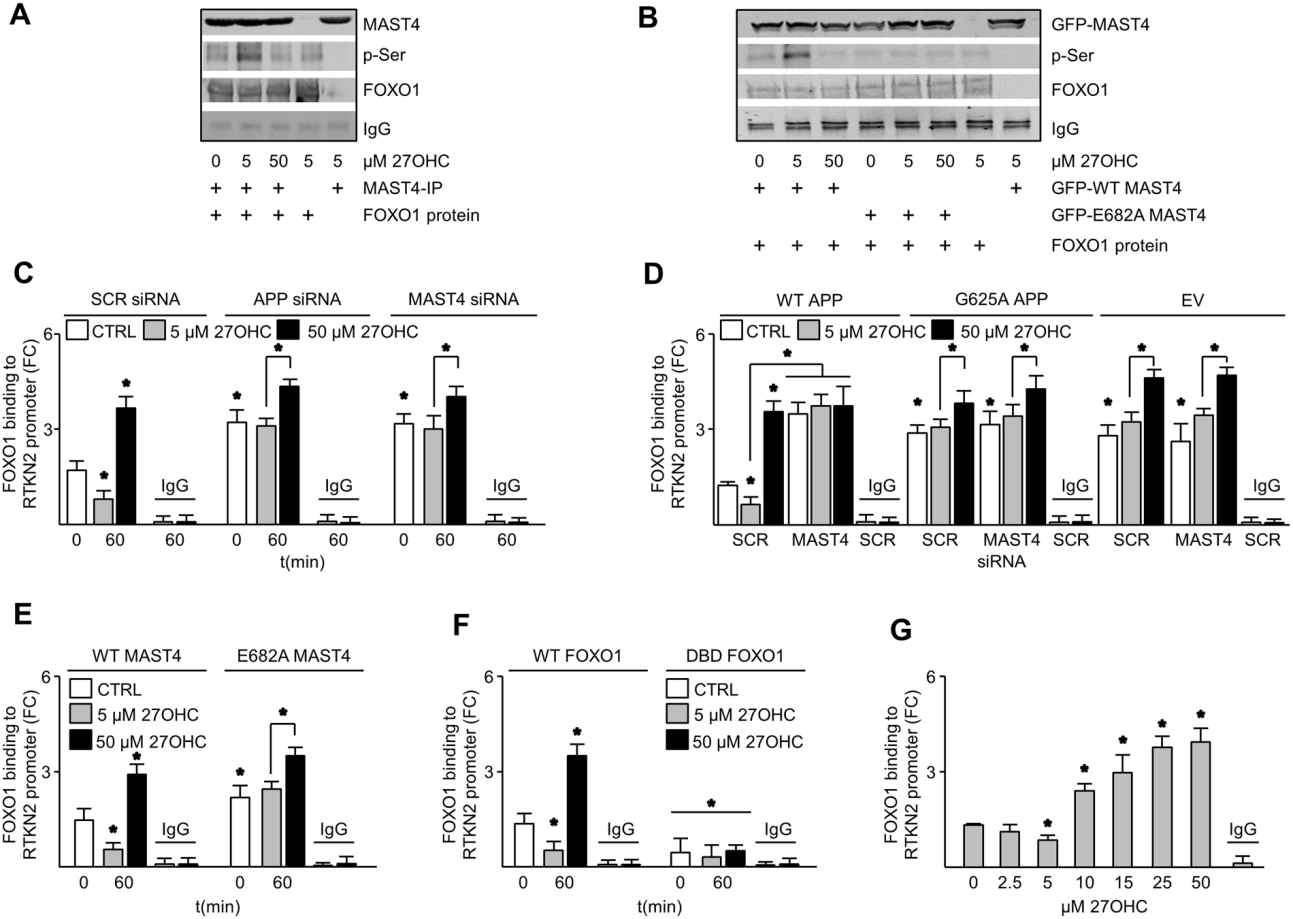
MAST4 phosphorylated and decreased FOXO1 binding to the *RTKN2* promoter. (**A**) Immunoblotting from kinase assay containing recombinant FOXO1 and immunoprecipitated MAST4 from nd-SH-SY5Y cells treated with 27OHC for 30 minutes or left untreated. (**B**) Immunoblotting from kinase assay containing recombinant FOXO1 and GFP tagged WT or E628A MAST4 immunoprecipitated from transfected nd-SH-SY5Y cells and treated as indicated for 30 minutes. FOXO1 binding to the *RTKN2* promoter in nd-SH-SY5Y cells transfected with SCR, APP, or MAST4 siRNA (**C**), B103 cells transfected with WT, APP G625A, or EV in combination with SCR or MAST4 siRNA (**D**), nd-SH-SY5Y cells transfected with WT or E682A MAST4 (**E**) or with WT or DBD FOXO1 (**F**), or RC neurons (**G**), and treated as indicated. ChIP and mRNA abundance are represented as fold change measurements (FC). (**A–G**) N = 3 independent experiments. * P < 0.05 significance is in comparison to the untreated control group of the SCR siRNA (**C**), SCR siRNA with WT APP (**D**), WT MAST4 (**E**), or WT FOXO1 (**F**) transfected cells or as indicated. (**G**) Significance is in comparison to 0 µM 27OHC treatment.

To establish a functional consequence of AICD transactivation of MAST4, kinase assays were conducted to determine if MAST4 can phosphorylate FOXO1. MAST4 increased the phosphorylation of FOXO1 in reactions containing immunoprecipitated MAST4 from lysates of nd-SH-SY5Y cells treated with 5 but not 0 or 50 µM 27OHC (Fig. 3A). To assess whether the predicted kinase domain of MAST4 was necessary for FOXO1 phosphorylation, wild-type glutamic acid 682, adjacent to the Walker-B motif, was substituted with alanine^19,20^. Immunoprecipitated E682A MAST4 did not phosphorylate FOXO1 regardless of the dose of 27OHC (Fig. 3B). In summary, these results demonstrate that MAST4 can phosphorylate FOXO1 but requires the glutamic acid residue adjacent to the Walker B motif.

Next, chromatin immunoprecipitation was performed to verify that FOXO1 binds to the *RTKN2* promoter and to determine how MAST4 affects FOXO1 activity. Five µM 27OHC decreased FOXO1 binding to the *RTKN2* promoter in nd-SH-SY5Y cells but not upon APP or MAST4 knockdown (Fig. 3C, S3A). Fifty µM 27OHC, however, increased FOXO1 binding to the RTKN2 promoter (Fig. 3C). AICD was not observed to directly bind to the RTKN2 promoter (Fig. S3F). Expression of WT APP, but not G625A APP, in B103 cells decreased FOXO1 binding to the *RTKN2* promoter (Fig. 3D). The same effect was observed in WT, but not E682A, MAST4 transfected nd-SH-SY5Y cells (Fig. 3E). Transfecting a FOXO1-DNA binding domain deletion mutant (DBD FOXO1)^12^ into nd-SH-SY5Y cells abrogated FOXO1 binding to the *RTKN2* promoter (Fig. 3F). Finally, the dose-dependent effect of 27OHC on this binding was confirmed in RC neurons (Fig. 3G). In summary, these results demonstrate that acting downstream to APP, MAST4 inhibits FOXO1 binding to the *RTKN2* promoter in response to 5 but not 50 µM 27OHC.

**Figure 4:**
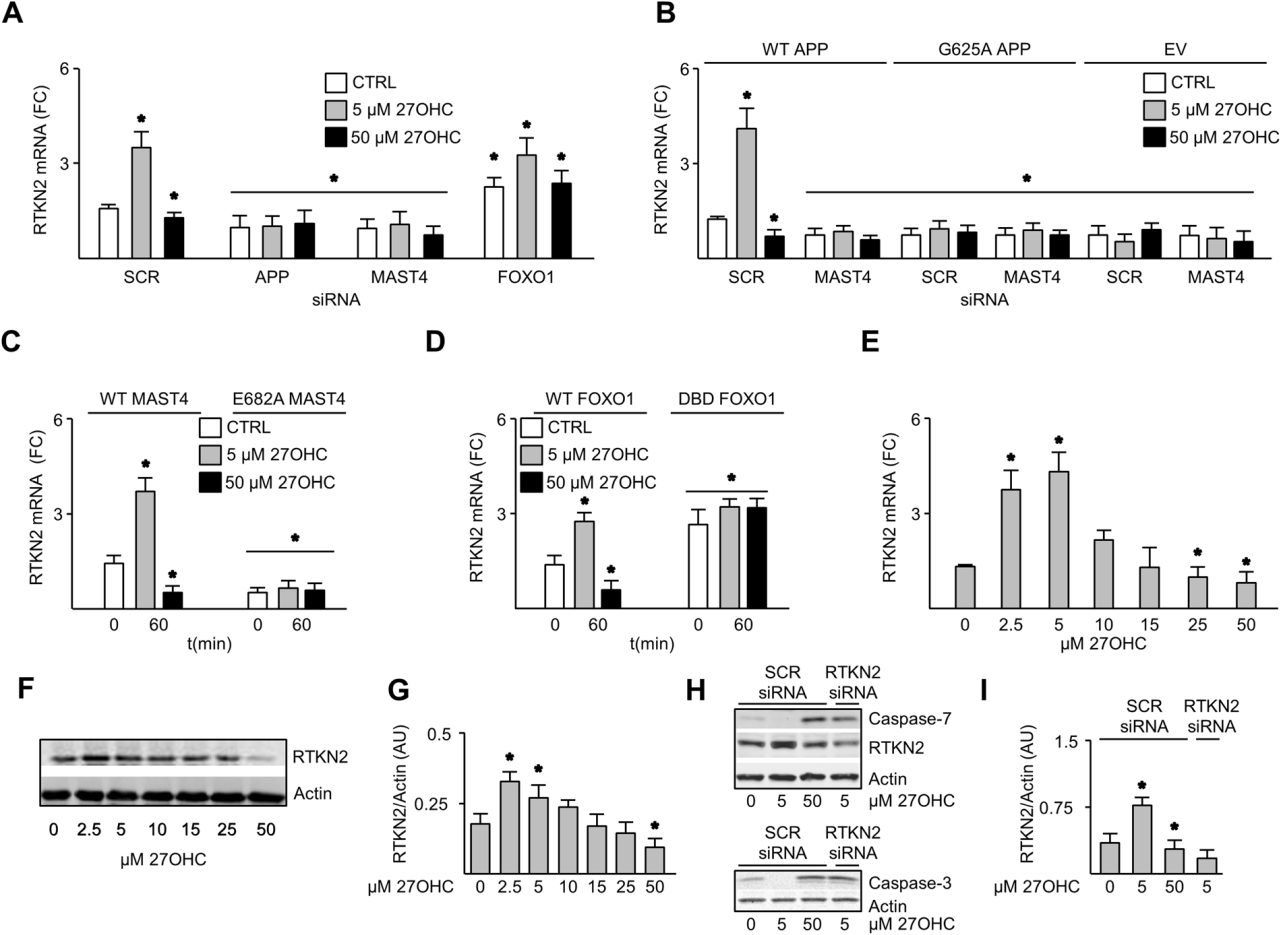
27OHC increased RTKN2 expression through APP, MAST4, and FOXO1. RTKN2 mRNA abundance in nd-SH-SY5Y cells transfected with SCR, APP, MAST4, or FOXO1 siRNA (**A**), B103 cells transfected with WT APP, G625A APP, or EV in combination with SCR or MAST4 siRNA (**B**), nd-SH-SY5Y cells transfected with WT or E628A MAST4 (**C**) or WT or DBD FOXO1 (**D**), or RC neurons (E), and treated as indicated. Immunoblotting (**F**) and quantification (**G**) of RTKN2 from RC neurons treated as indicated. Immunoblotting (**H**) and quantification (**I**) of caspases-7 and -3 and RTKN2 in nd-SH-SY5Y cells transfected with SCR or RTKN2 siRNA and treated as indicated. ChIP and mRNA abundance are represented as fold change measurements (FC). (**A–I**) N = 3 independent experiments. *P < 0.05 significance is in comparison to the untreated control group of the SCR siRNA (**A**), SCR siRNA with WT APP (**B**), WT MAST4 (**C**), or WT FOXO1 (**D**) transfected cells. (**E** & **G**) Significance is in comparison to 0 µM 27OHC treatment. (**I**) Significance is in comparison to the untreated control group of the SCR siRNA transfected cells.

Next, the effect of 27OHC on RTKN2 expression was determined. RTKN2 mRNA abundance increased in nd-SH-SY5Y cells treated with 5 and inhibited by 50 µM 27OHC. This signaling event was not observed following APP or MAST4 knockdown (Fig. 4A). RTKN2 mRNA levels increased in cells transfected with FOXO1 siRNA irrespective of the 27OHC dose (Fig. 4A). Five micromolar 27OHC increased while 50 µM 27OHC inhibited RTKN2 mRNA induction in B103 cells transfected with WT, but not G625A, APP (Fig. 4B). Similar results were seen in WT, but not E682A MAST4 transfected nd-SH-SY5Y cells (Fig. 4C). RTKN2 mRNA increased in DBD FOXO1 expressing nd-SH-SY5Y cells irrespective of 27OHC dose (Fig. 4D). The dose-dependent effect of 27OHC on RTKN2 transcription and protein level was confirmed in RC neurons (Fig. 4E–G). Five µM 27OHC increased RTKN2 and decreased active caspases 3 and 7 protein abundance, but not when RTKN2 was knocked down (Fig. 4H–I); demonstrating that its expression is necessary for the observed cytoprotective response to 27OHC. Taken together, these data validate the key elements of the proposed molecular model (Fig. 1J) that responds hormetically to 27OHC doses.

### APP governs RTKN2 expression through MAST4 and FOXO1 ***in vivo***

**Figure 5:**
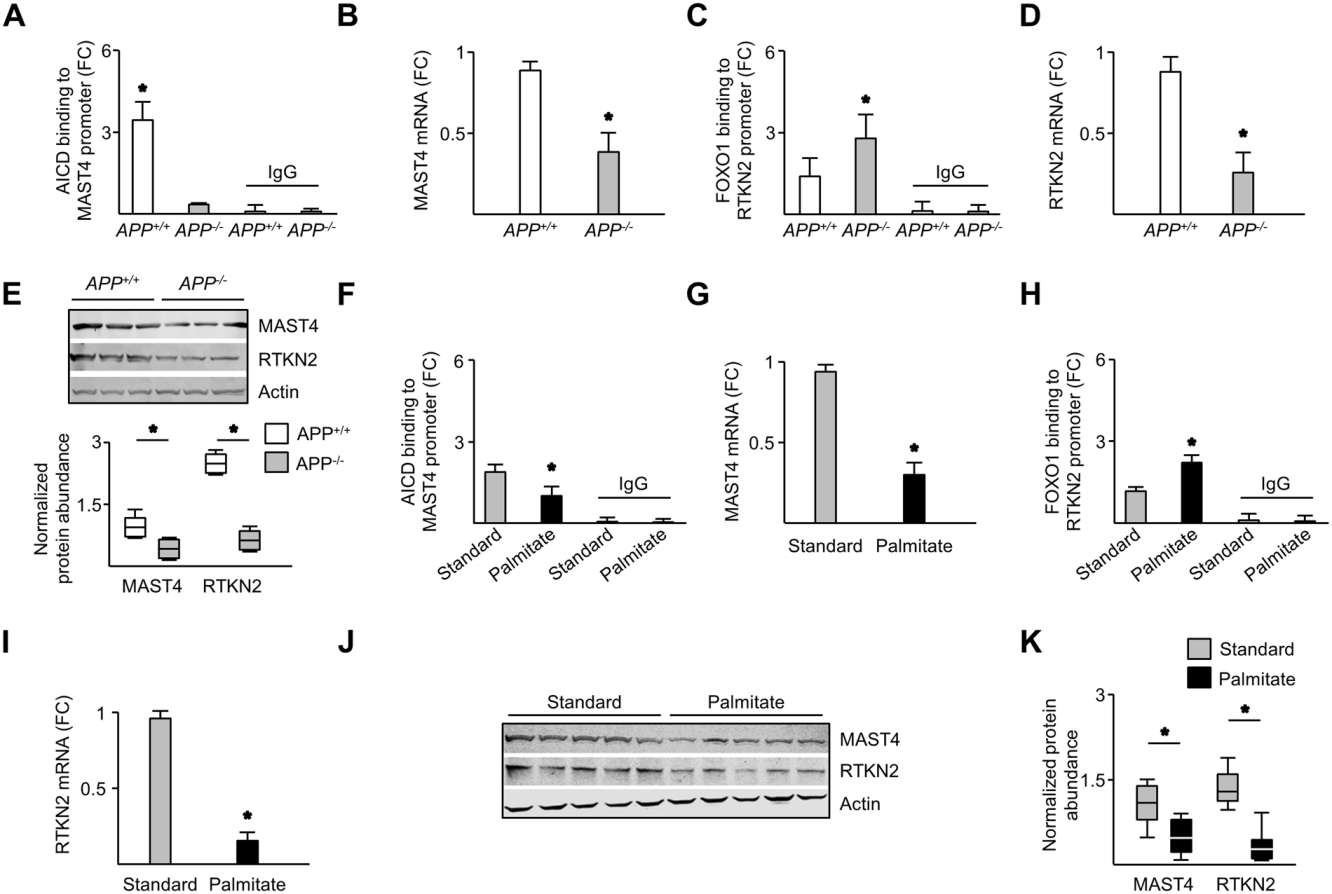
APP ablation and palmitate-rich diet decreased AICD/MAST4/FOXO1/RTKN2 signaling *in vivo*. AICD binding to *MAST4* promoter (**A**), MAST4 mRNA abundance (**B**), FOXO1 binding to the RTKN2 promoter (**C**), RTKN2 mRNA abundance (**D**), and immunoblotting of MAST4 and RTKN2 (upper panel) with quantification (lower panel) (**E**), in APP^+/+^ and APP^*−*/*−*^ mouse cortical samples. (**A–E**) N = 3 independent experiments. AICD binding to the *MAST4* promoter (**F**), MAST4 mRNA abundance (**G**), FOXO1 binding to the *RTKN2* promoter (**H**), RTKN2 mRNA abundance (**I**), and immunoblotting of MAST4 and RTKN2 (**J**) and quantification (**K**), from the cortices of mice fed standard or palmitate diet. ChIP and mRNA abundance are represented as fold change measurements (FC). (**F–K**) N = 5 independent experiments. *P < 0.05 significance is in comparison to the APP^*−*/*−*^ genetic background (**A**), APP^+/+^ genetic background (**B–E**), or standard diet (**F**–**I** and **K**).

To determine if APP is important for the basal activation of the AICD-MAST4-FOXO1-RTKN2 pathway, cerebral cortices from *APP* ^+/+^ mice and *APP* ^*−/−*^ littermates fed a normal diet, mimicking 5 µM 27OHC, were used. Higher AICD binding to the *MAST4* promoter and MAST4 mRNA abundance was observed in *APP* ^+/+^ relative to *APP* ^*−/−*^ brains (Fig. 5A,B). Further, FOXO1 binding to the *RTKN2* promoter was lower and RTKN2 mRNA was higher in *APP* ^+/+^ brains compared to *APP* ^*−/−*^ brains (Fig. 5C,D). Finally, both MAST4 and RTKN2 and protein abundance were higher in the *APP* ^+/+^ brains compared to *APP* ^*−/−*^ brains (Fig. 5E). These findings indicate that AICD-MAST4-FOXO1-RTKN2 signaling is intact *in vivo*.

The pro-inflammatory, palmitic acid-rich diet, increases 27OHC and has been shown to induce a cognitively impaired phenotype^24,25^. To determine whether the AICD-MAST4-FOXO1-RTKN2 response can be affected by diet, four-week old *APP* ^+/+^ mice were fed a normal or palmitic acid-rich diet for 16 weeks and cerebral cortices removed. Cerebral cortices from *APP* ^+/+^ mice fed the palmitic acid-rich diet had less AICD association with the *MAST4* promoter (Fig. 5F); less MAST4 mRNA (Fig. 5G) abundance; more FOXO1 association to the *RTNK2* promoter (Fig. 5H); and less RTKN2 mRNA abundance (Fig. 5I). MAST4 and RTKN2 protein abundance was also lower in *APP* ^+/+^ mice fed the palmitic acid-rich diet (Fig. 5J,K). These data highlight the negative effect a high palmitic diet has on this cytoprotective pathway.

### AICD-driven regulation of RTKN2 is impaired in late-onset AD but not in frontotemporal dementia

**Figure 6:**
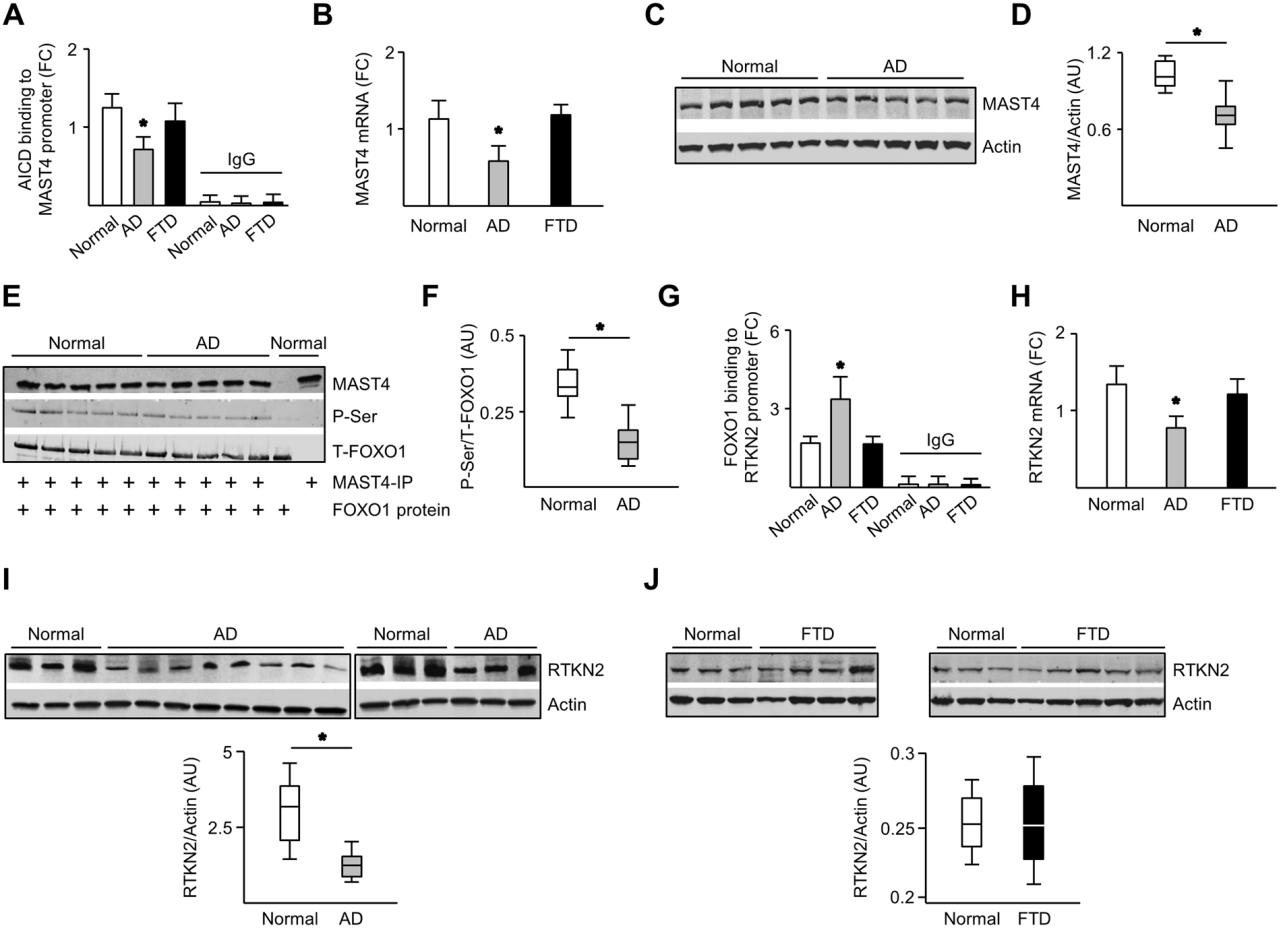
The AICD/MAST4/FOXO1/RTKN2 pathway is repressed in AD but not FTD. AICD binding to the *MAST4* promoter (**A**), MAST4 mRNA abundance (**B**), and Immunoblotting of MAST4 (**C**) with quantification (**D**) of temporal lobe samples from AD and FTD patients. Immunoblotting from kinase assay (**E**) and quantification (**F**) containing recombinant FOXO1 and immunoprecipitated MAST4 from temporal lobe samples from patients with normal cognitive function or AD. CTRL reactions contain a FOXO1 only control or MAST4 immunoprecipitation only from a representative control patient. FOXO1 binding to the *RTKN2* promoter (**G**) and RTKN2 mRNA abundance (**H**) in the temporal lobe from AD and FTD patients. Immunoblotting (upper panels) and quantification (lower panels) of RTKN2 from temporal lobe samples from patients with AD (**I**), FTD (**J**), or normal cognitive function. Panel I and J contain the same three Normal samples loaded in each gel. ChIP and mRNA abundance are represented as fold change measurements (FC). (**A–F**) N = 5 samples. (**G–J**) normal N = 3 samples, AD N = 11 samples, FTD N = 9 samples. *P < 0.05 significance is in comparison to normal samples.

Given that late-onset AD is often associated with cholesterol dysregulation and dyslipidemia^10^, assays were performed to determine if the AICD-MAST4-FOXO1-RTKN2 pathway is perturbed in late-onset AD brains. On the other hand, frontotemporal dementia is associated with genetic risk factors and unknown environmental causes^14^. Therefore, normal brains and FTD brains were assessed as a control and comparison respectively. AICD binding to the *MAST4* promoter was lower in late-onset AD brains compared to control cases (no neurologic disease) and FTD brains (Fig. 6A), which was accompanied by less MAST4 mRNA and protein abundance (Fig. 6B–D). In vitro kinase activity performed with immunoprecipitated MAST4 from late-onset AD brains displayed lower intrinsic kinase activity on recombinant FOXO1 when compared to normal brains (Fig. 6E,F). FOXO1 binding to the RTKN2 promoter was increased (Fig. 6G); while RTKN2 mRNA and protein levels were lower in late-onset AD brains compared to normal and FTD brains (Fig. 6H–J). These results elucidate a novel APP regulated cytoprotective pathway in normal and FTD brains that is not active in the late-onset AD brains.

## Discussion

Crossing the blood brain barrier, 27OHC initiates a myriad of homeostatic functions including regulation of neuronal morphology, lipid metabolism, and sterol-dependent neuronal signaling^26^. Hormesis occurs in response to an array of stress stimuli, including oxidative stress and inflammation in the brain, differentiating between normal and aberrant physiological states^16,27,28^. This study demonstrates that the cytoprotective role of low, but not high, 27OHC doses requires a sterol-sensing domain of APP^22,29,30^ to liberate AICD. Increased 27OHC biogenesis may trigger increased oxidative stress resulting in a molecular switch rearranging the cellular homeostatic functions of APP and AICD culminating in neuronal pathogenesis (Fig. 6).

Although the exact mechanism by which APP is processed in response to 27OHC is still unknown, it is likely that this is a multi-tiered process promoted by direct 27OHC binding to APP, inducing conformational changes and liberating AICD^20^. Additionally, AICD liberation could occur by increasing the activity of secretases, such as β-secretase known to be activated by 27OHC^17^. G625 in APP_695_ isoform, equivalent to G700 in isoform APP_770_, resides at the extracellular membrane interface located in tandem GXXG motifs that are important for cholesterol binding^22^. Glycine to alanine mutation disrupts van der Waals interactions with sterols hampering APP cholesterol binding^22^. Therefore, G625A APP likely attenuates association with cholesterol-rich lipid rafts (Fig. S3E); impairs 27OHC binding-induced structural changes and APP cleavage; and AICD-mediated transcription on a genome-wide scale (Figs 2D, 3D and 4B)^22^. These impairments may explain the attenuation of AICD binding to the MAST4 promoter in response to 50 µM 27OHC (Fig. 2A).

The duality of AICD function, in the context of neuronal health and AD development, is consistent with a hormetic response^29–31^. For example, AICD serves beneficial effects by increasing the amyloid-β peptide degrading enzyme neprilysin (NEP). Yet AICD also promotes deleterious effects in the context of AD by increasing the expression of Tau-phosphorylating glycogen synthase kinase 3 beta (GSK3β), the amyloidogenic β-secretase 1 (BACE1), the pro-apoptotic protein p53, and indirectly decreases the expression of the non-amyloidogenic α-secretase ADAM Metallopeptidase Domain 10 (ADAM10)^32–36^. In response to low concentrations of 27OHC, AICD transactivated MAST4 and increased its activity but not at high concentrations (Figs 2 and 3). These results suggest that AICD binding partners, or lack thereof, direct its genome specificity, which is dependent upon the relative degree and type of upstream signaling^13,31^, and may determine its beneficial or deleterious effects. Similarly, MAST4 activity is altered in response to 5 and 50 µM 27OHC in kinase reactions containing normalized protein abundance (Figs 3A,B, 6E). This suggests that in addition to transcriptional activation, 5 µM 27OHC induces posttranslational regulation of MAST4 to increase its kinase activity.

MAST4 is emerging as a neuroprotective mediator, exemplified by genome-wide association studies linking MAST4 mutations to abnormalities in hippocampal anatomy^37^ and myoclonic epilepsy^38^, an accepted symptom of AD^39^. MAST4 is particularly important in oligodendrocytes^37^. Dysfunctional oligodendrocytes induce myelin breakdown, which is associated with AD^40,41^. Although evidence demonstrates the relevance of MAST4 to the hippocampus, a region whose neuronal architecture is essential for memory formation, data illustrated in Fig. 6 suggest it plays an important role in temporal regions of the brain to prevent AD pathogenesis^41,42^.

This report is the first to provide evidence for MAST4 as a kinase. The catalytic domain of MAST4 contains both a walker B motif that creates a hydrophobic environment for ATP binding and an N-terminal acidic residue (glutamic acid) that facilitates hydrolysis of ATP, terminal phosphate transfer, and electrostatic interactions to align catalytic residues (Fig. 1G and S1)^43,44^. These data indicate that G682A mutagenesis on MAST4 disrupts its kinase activity on FOXO1 (Fig. 3B).

FOXO1 serves as a homeostatic switch for neuronal health, differentiation and survival depending on expression and upstream signaling^45^. For example, posttranslational regulation (deacetylation) of FOXO1 prevents inflammation-induced apoptosis;^46^ while high oxidative stress elicits an AICD-FOXO1 complex that transactivates the Bcl-2-like protein 11 (BIM), inducing apoptosis^13^. However, under low concentrations of 27OHC, phosphorylation of FOXO1 by MAST4 decreased transcriptional repression of RTKN2 rendering an anti-apoptotic phenotype (Figs 3 and 4).

Although there are genetic factors in the causation of neurodegenerative disorders, such as AD, life style and diet have emerged as independent causative factors, particularly diets that promote dyslipidemia and cholesterol dysregulation. Obesogenic diets rich in palmitic acid, the most abundant fatty acid in typical Western diets, lead to cognitive and behavioral impairment in mice, and are associated with higher risk of late onset AD^25,26^. This study demonstrates that a pro-inflammatory, palmitate-rich diet inhibits the AICD-MAST4-FOXO1-RTKN2 driven neuronal protective signaling in mice brains in an APP-dependent manner (Fig. 5). Illustrated in Figs 1–4, APP and MAST4 function to optimize cellular survival under normal homeostatic conditions in vitro with an optimal cellular response at 5 µM 27OHC. In vivo, 50 µM 27OHC may correlate with biochemical alterations observed in palmitate fed mice (Fig. 5). As complete depletion to 27OHC is not physiological *in vivo*, 5 µM 27OHC in vitro doses may correspond to normal *in vivo* physiology while elevated levels correlate to pathophysiology. Moreover, this cytoprotective pathway was functional in normal and FTD human brains but attenuated in AD human brains (Fig. 6). These results offer a molecular rationale for labeling a diet that induces cholesterol dysregulation and dyslipidemia as a modifiable risk factor for neurodegeneration, and it provides insight toward therapeutic approaches against cholesterol-associated neuronal disorders.

## Materials and Methods

### Bioinformatics and data mining

Entrosolve (Entrosolve.com) was recruited to mine all large datasets, conduct consensus sequence mapping, and identify signaling pathways.

### Cell isolation and culture

Rat cortical neurons were dissociated using a papain dissociation kit following manufacturer’s instructions (Worthington, NJ; Cat# LK003150). Neurons were cultured in neurobasal medium with B27 supplement with 2 mM glutamine, 50 U/mL penicillin and 50 μg/mL. SH-SY5Y and B103 cells were cultured in DMEM (Sigma Cat # D6429–500M) supplemented with 5% fetal bovine serum. SH-SY5Y cells were differentiated with the addition of 10 µM retinoic acid for 7 days prior to experimentation.

### Transfection

Transfections were conducted using Lippofectamine LTX (Thermofisher; cat# A12621) according to the manufacturer’s instructions.

### Human brains

Postmortem tissue was obtained from the Easton Alzheimer’s Disease Research Center Brain Bank at the University of California, Los Angeles. Diagnoses were established using accepted clinical and histopathologic criteria.

All patients and/or their legal guardians gave their informed consent to participate in research protocols prior to tissue donation.

All methods and protocols, including those necessary to ensure the privacy rights of human subjects, were carried out in accordance with relevant institutional regulations and were approved by Institutional Review Board of Loma Linda University Medical Center (approval #54174).

### Animal studies

All animal procedures were carried out in accordance with the U.S. Public Health Service Policy on the Humane Care and Use of Laboratory Animals and were approved by the Institutional Animal Care and Use Committee at the University of North Dakota (Protocol 1506-3c). All animal experiments comply with the National Institutes of Health guide for the care and use of Laboratory animals (NIH Publications No. 8023, revised 1978). The mice were housed in individually ventilated cages at an ambient room temperature (23–25 °C) and ambient relative humidity ranging between 50 and 70%. The mice were maintained on 12:12 h light: dark cycle. Male C57BL/6 J mice (6-week-old) were fed a normal or palmitate-enriched diet for 16 weeks (n = 6 per group). The normal diet contains 0.8% palmitate and 2.2% linoleic acid (NIH-07 open Formula Mouse, TD. 8 + 5172; Herlan Teklad). The palmitate-enriched diet is formulated by adding 30 g/kg palm oil (3%) to NIH-07 mice diet to increase the palmitic acid from 0.8 to 2.2% by weight and lowering linoleic acid from 2.2% to 0.8% (TD.110616, Harlan Teklad). Control and palmitate diets are isocaloric, the key difference residing in the palmitate levels.

### Microarray Transcriptional Profiling

Mice used in this study to generate microarray transcriptomes have been described in detail^9^. Samples were flash-frozen in liquid nitrogen, and frozen samples sent to Genus Biosystems (Northbrook, Il) for Micro Array transcriptional profiling.

### RNA purification

RNA was purified using TRIzol LS reagent according to the manufacturer’s instructions (Thermofisher cat# 10296010).

### Chromatin Immunoprecipitation Assays

Chromatin Immunoprecipitation (ChIP) assays were conducted according to standard protocols published by ABcam. Following treatments, cells were incubated with formaldehyde at a final concentration of 0.75% for 10 minutes followed by glycine at a final concentration of 125 mM for an additional 5 minutes. Cells were then washed two times with Phosphate Buffered Saline (PBS) and lysed in FA lysis buffer (50 mM HEPES-KOH pH 7.5, 140 mM NaCl, 1 mM EDTA, 1% Triton X-100, 0.1% Sodium Deoxycholate, 0.1% SDS, protease inhibitors). Resulting cell lysates were sonicated to fragment DNA, spun down, and incubated with antibody conjugated protein A sepharose beads (ThermoFisher cat# 101041) overnight with gentile agitation. Following incubation, beads were pelleted, washed three times with FA lysis buffer, and DNA was eluted with elution buffer (1% SDS, 100 mM NaHCO_3_). Resulting DNA fragments were further purified with a DNA purification kit (Clontech cat# 740609.250) prior to qPCR analysis. Antibodies used were anti-APP C-terminus (Sigma #A8717); anti-FOXO1 (Abcam #39670). Primer sequences listed in Table S1.

### qPCR

mRNA was purified with TRIzol reagent, converted to cDNA with reverse transcriptase according to the manufacturer’s instructions and quantified with iTaq Universal SYBR Green Supermix (Bio-Rad cat#1725120). Results were determined using the delta-delta cycle threshold (ct) method. Primer sequences listed in Table S1.

### Immunoblotting

Immunoblotting was conducted as previously described with the following antibodies: APP (Sigma #A8717; Millipore #22C11); MAST4 (GeneTex #GTX87899); FOXO1 (Cell Signaling Technology #2880); RTKN2 (Proteintech #17458-1-AP); caspase-3 (Cell Signaling Technology #9662); caspase-7 (Cell Signaling Technology #9492); actin (Sigma #A5316)^7^.

### Immunoprecipitation

Immunoprecipitations were conducted as outlined by ABcam under non-denaturing conditions.

### MAST4 in vitro kinase assay

Kinase activity was measured at 37 °C for 30 minutes in 50 µl kinase buffer (50 mM Tris, pH 7.4, 10 mM MgCl2) supplemented with 50 µM ATP and human recombinant FOXO1 (1 µg; Origene #TP300477). Kinase reactions were run on 4–20% Tris-Glycine polyacrylamide gels (Thermofisher) and byproducts identified with anti phospho-Serine/Threonine antibody (Abcam ab17464).

### LDH assay

LDH assays were conducted with a LDH assay kit following manufacturer’s instructions (cat# 88954).

### Live Dead cell assay

Live dead cell assays were conducted with live dead cell assay kit according to the manufacturer’s instructions (Thermofisher# L3224).

### Lipid raft fractionation

Lipid rafts were isolated using a detergent-free method. Specifically, cells were grown to 80% confluence in 10 cm dishes, washed twice with ice cold PBS before being lysed with 2 ml of 100 mM Na2CO3, pH 11.0 plus HaltTM protease and phosphatase inhibitor cocktail (Thermofisher cat#78443). The cell suspension was homogenised with 8 strokes of a Dounce homogeniser and then sonicated using continuous sonication with a Vibra Cell (Sonics and Materials, USA) on power setting 1 (3 × 20 s). The homogenate was then adjusted to 45% sucrose by mixing with 2 ml of 90% (w/v) sucrose solution in MBS buffer and then added to a 12 ml Beckman ultrclear centrifuge tube. 4 ml of 35% (w/v) sucrose was carefully layered on top, followed by 4 ml of 5% (w/v) sucrose solution. 90% (w/v) sucrose solution was prepared in MBS buffer (25 mM Mes, 150 mM NaCl, pH 6.5). Both the 35% and 5% sucrose solutions were prepared in MBS buffer plus 250 mM Na2CO3. The samples were then centrifuged at 175000 g (39000 rpm using a Beckman SW41 rotor) for 18 h at 4 °C. 1 ml fractions were taken from the top of the tube and stored at −80 °C.

### Statistical Analysis

Data are means ±SEM of at least three independent experiments. Tests used for nonparametric data included Kruskal-Wallis test with Tukey’s post hoc test and Mann-Whitney U test. Parametric data were analyzed using analysis of variance (ANOVA) with post hoc Bonferroni. Unless otherwise indicated, P values < 0.05 are considered statistically significant.

### Data Availability Statement

The datasets generated and analysed during the current study are available from the corresponding author on reasonable request.

### One Sentence Summary

27OHC incites cytoprotection through ACID transactivation of MAST4, which phosphorylates FOXO1 to increase RTKN2.

## Electronic supplementary material

Supplementary materials
