# Supplementary material for: Cellular hormetic response to 27-hydroxycholesterol promotes neuroprotection through AICD induction of MAST4 abundance and kinase activity
Source: Sci Rep. 2017 Oct 24;7:13898. doi: 10.1038/s41598-017-13933-9 (PMC5654999; doi:10.1038/s41598-017-13933-9)
Supplement: Supplementary file 1 — Supplementary materials [file 41598_2017_13933_MOESM1_ESM.pdf]

**Title:**

# Cellular hormetic response to 27-hydroxycholesterol promotes neuroprotection through AICD induction of MAST4 abundance and kinase activity

**Authors:**

Brendan Gongol[1,2**], Traci L Marin[2**], John D Jeppson[1], Karina Mayagoitia[1], Samuel Shin[1], Nicholas Sanchez[3], Wolff M. Kirsch[3], Harry V. Vinters[4], Christopher G. Wilson[5], Othman Ghribi[6], Salvador Soriano[1,*]

**Affiliations:**

[1]Department of Pathology and Human Anatomy, Division of Anatomy, Loma Linda University School of Medicine, Loma Linda CA 92350, USA.

[2]Cardiopulmonary Sciences, Schools of Allied Health Professions and Medicine, Loma Linda University, Loma Linda CA 92350, USA.

[3]Department of Basic Sciences, Division of Biochemistry, School of Medicine, Loma Linda University, Loma Linda CA 92350, USA.

[4]Section of Neuropathology, Ronald Reagan UCLA Medical Center, David Geffen School of Medicine at UCLA, Los Angeles 90095, USA.

[5]Department of Basic Sciences, Division of Physiology, School of Medicine, Loma Linda University, Loma Linda CA 92350, USA.

[6]Department of Basic Sciences, School of Medicine and Health Sciences, University of North Dakota, Grand Forks, ND 58202, USA.

*Correspondence to: ssoriano@llu.edu; Phone # 909-588-7010

**Authors contributed equally.

**Running title:** 27OHC incites cytoprotection through ACID transactivation of MAST4, which phosphorylates FOXO1 to increase RTKN2.

**A**

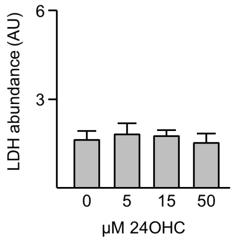


**B**

```
HUMAN DFETIKLISNGAYGAVYFVRHKESRQRFAMKKINKQNLILRNQIQQAFVERDILTFAENPFVVSMYCSFETRRHLCMVMEYVEGGDCATLMK
RAT   DFETIKLISNGAYGAVYFVRHKESRQRFAMKKINKQNLILRNQIQQAFVERDILTFAENPFVVSMYCSFETRRHLCMVMEYVEGGDCATLMK
MOUSE DFETIKLISNGAYGAVYFVRHKESRQRFAMKKINKQNLILRNQIQQAFVERDILTFAENPFVVSMYCSFETRRHLCMVMEYVEGGDCATLMK

HUMAN NMGPLPVDMARMYFAETVLALEYLHNYGIVHRDLKPDNLLVTSMGHIKLTDFGLSKVGLMSMTTNLYEGHIEKDAREFLDKQVCGTPEYIAP
RAT   NMGPLPVDMARMYFAETVLALEYLHNYGIVHRDLKPDNLLVTSMGHIKLTDFGLSKVGLMSMTTNLYEGHIEKDAREFLDKQVCGTPEYIAP
MOUSE NMGPLPVDMARMYFAETVLALEYLHNYGIVHRDLKPDNLLVTSMGHIKLTDFGLSKVGLMSMTTNLYEGHIEKDAREFLDKQVCGTPEYIAP

HUMAN EVILRQGYGKPVDWWAMGIILYEFLVGCVPFFGDTPEELFGQVISDEINWPEKDEAPPPDAQDLITLLLRQNPLERLGTGGAYEVKQHRFF
RAT   EVILRQGYGKPVDWWAMGIILYEFLVGCVPFFGDTPEELFGQVISDEINWPEKDEAPPPDAQDLITLLLRQNPLERLGTGGAYEVKQHRFF
MOUSE EVILRQGYGKPVDWWAMGIILYEFLVGCVPFFGDTPEELFGQVISDEINWPEKDEAPPPDAQELITLLLRQNPLERLGTGGAYEVKQHRFF
```

**C**

```
HUMAN                    DMARMYFAETVLALEYLHNYGIVHR
MOUSE                    DMARMYFAETVLALEYLHNYGIVHR
RAT                      DMARMYFAETVLALEYLHNYGIVHR
ZEBRAFISH                DMARMYFAETVLALEYLHNYGIVHR
CHICKEN                  DMARMYFAETVLALEYLHNYGIVHR
CHIMPANZEE               DMARMYFAETVLALEYLHNYGIVHR
DOG                      DMARMYFAETVLALEYLHNYGIVHR
CATTLE                   DMARMYFAETVLALEYLHNYGIVHR
FROG                     DMARMYFAETVLALEYLHNYGIVHR
BALD EAGLE               DMARMYFAETVLALEYLHNYGIVHR
PHILLIPINE TARSIER       DMARMYFAETVLALEYLHNYGIVHR
BOLIVIAN SQUIRREL MONKEY DMARMYFAETVLALEYLHNYGIVHR
POSSUM                   DMARMYFAETVLALEYLHNYGIVHR
TASMANIAN DEVIL          DMARMYFAETVLALEYLHNYGIVHR
```

**Supplemental Fig S1. Effects of 24OHC on LDH production and MAST4 kinase domain sequence conservation.** (A) Effects of 24OHC on LDH production. (B) MAST4 kinase domain sequence and (C) catalytic domain conservation.

**A**

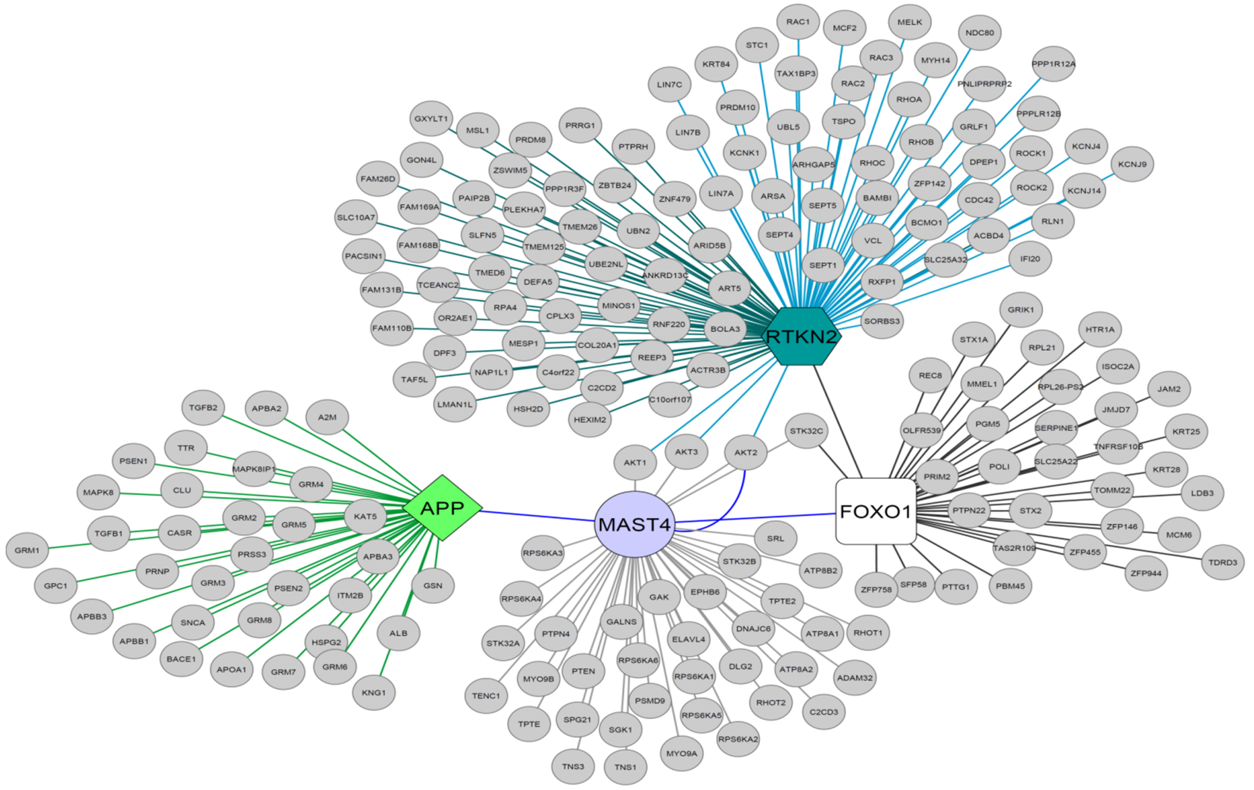


**Supplemental Fig S2. APP, MAST4, FOXO1, RTKN2 nodal connections and supporting data for siRNA efficiency.** (A) APP, MAST4, FOXO1, RTKN2 nodal connections.

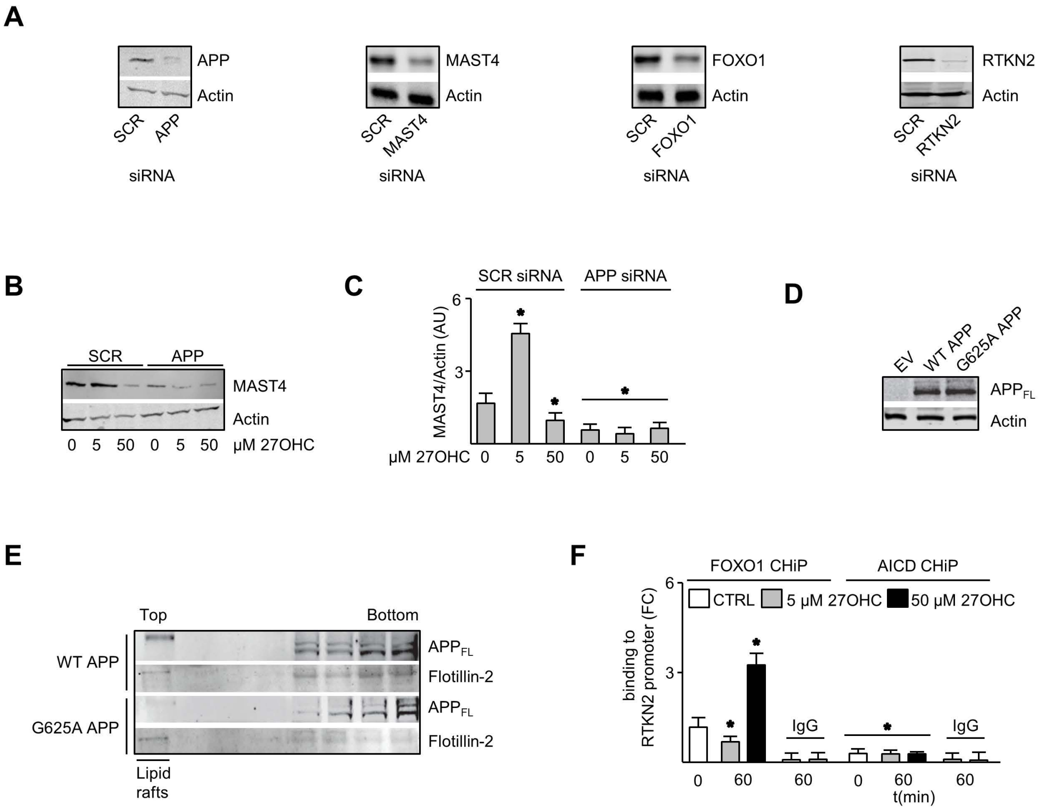


**Supplemental Fig S3. APP trafficking and AICD RTKN2 binding supporting data.** (A) Immunoblots demonstrating relative siRNA efficiency. MAST4 protein * abundance (B) and quantification (C) in nd-SH-SY5Y cells transfected with SCR or APP siRNA and treated with 5 or 50 µM 27OHC for 60 minutes. (D) Protein abundance in B103 cells transfected with EV, WT APP, or G625 APP. (E) lipid raft fractionation in cells transfected with WT or G625 APP. (F) FOXO1 or AICD CHiP in cells treated with 0, 5, or 50 µM 27OHC. ChIP abundance is represented as fold change measurements (FC). (A-F) N=3 independent experiments. * $P < 0.05$ significance is in comparison to the untreated control group in the SCR siRNA transfected cells (C) or to the FOXO1 CHiP CTRL group (F).

| Human mRNA Primers | | |
|---|---|---|
| | **Forward Sequence** | **Reverse Sequence** |
| **RTKN2** | CCAGAGGAAATTGAAGCTAAAGTG | TGTCCAGGAACAGGATTGATG |
| **MAST4** | GTGGAATTGCTTGGTCAAACG | ACTGATGCAACTTCTCCTGG |
| **β-Actin** | CATGTACGTTGCTATCCAGGC | CTCCTTAATGTCACGCACGAT |

| Mouse mRNA Primers | | |
|---|---|---|
| | **Forward Sequence** | **Reverse Sequence** |
| **RTKN2** | CTTGGAAAATGCTGGAGACTG | GAGATCAAAGAAATGTTGCCGG |
| **MAST4** | AAAGTCACAAAGTCCCTCTCG | ACCTTATTCCCACTCTTCAGC |
| **β-Actin** | AGGCCGGTGCTGAGTATGTC | TGCCTGCTTCACCACCTTCT |

| Rat mRNA Primers | | |
|---|---|---|
| | **Forward Sequence** | **Reverse Sequence** |
| **RTKN2** | GAAAGCGGATATGTGAGAGGG | CACTCTAGCCGAATGTACTGG |
| **MAST4** | AGTCCATAAAGCGTCCAAGC | TTCTTGTAACTCCCATCCTGC |
| **β-Actin** | GGGAAATCGTGCGTGACATT | GCGGCAGTGGCCATCTC |

| Human Promoter Primers | | |
|---|---|---|
| | **Forward Sequence** | **Reverse Sequence** |
| **RTKN2** | GATATCGACCTTCTGTAAGAGCC | AGTTCCCAGAAAGTGAGAAGTAC |
| **MAST4** | CACAACTCACCTCTGATTCTCC | ACCCTACTCCTGCCTCTTAC |
| **β-Actin** | CGACCAGTGTTTGCCTTTTATG | ATGGTGAGCTGCGAGAATAG |

| Mouse Promoter Primers | | |
|---|---|---|
| | **Forward Sequence** | **Reverse Sequence** |
| **RTKN2** | CATCCTCAGCTACCACTCTTTAAG | AGAACCAGCCATCAACACG |
| **MAST4** | CTCCTGGGTACATCTCCTTTTG | CAAAAGGAGATGTACCCAGGAG |
| **GAPDH** | CCCTGTTCTCCCATTTTACTCG | GCTTATCCAGTCCTAGCTCAAG |

| Rat Promoter Primers | | |
|---|---|---|
| | **Forward Sequence** | **Reverse Sequence** |
| **RTKN2** | ATTTTCACCTCTTACCGGCTC | AGGACACCCAGAATACACAAC |
| **MAST4** | TCTGGGTATGCTAGGCTTAGG | AAGGACTATCTGATTGGCTGAC |
| **β-Actin** | GAGTGGTCAAGATCCCTGAAG | AGAGGATGAAGAGTTTGGCG |

**Table S1. Primer sequences used for ChIP and qPCR assays.**
